# Simulation Platform for SINS/GPS Integrated Navigation System of Hypersonic Vehicles Based on Flight Mechanics

**DOI:** 10.3390/s20185418

**Published:** 2020-09-21

**Authors:** Kai Chen, Fuqiang Shen, Jun Zhou, Xiaofeng Wu

**Affiliations:** 1 Northwestern Polytechnical University, Xi’an, Shanxi 710072, China; 2018260368@mail.nwpu.edu.cn (F.S.); zj2019@mail.nwpu.edu.cn (J.Z.); 2 School of Aerospace, Mechanical and Mechatronic Engineering, The University of Sydney, Sydney 2006, Australia; xiaofeng.wu@sydney.edu.au

**Keywords:** trajectory generator, hypersonic vehicle, SINS/GPS integrated navigation, hardware-in-the-loop simulation

## Abstract

In this study, a simulation platform for an integrated navigation algorithm for hypersonic vehicles based on flight mechanics is designed. In addition, the generation method of inertial measurement unit data and satellite receiver data is introduced. First, the interface relationship between a high-precision six-degree-of-freedom (6DoF) model and the simulation platform in the launch-centered Earth-fixed frame is introduced. Three-axis theoretical specific force and angular velocity are output by the 6DoF model. Accelerometer and gyroscope error models are added, and integral processing of the specific force and angular velocity is performed to obtain velocity increment of the accelerometer and the angular increment of the gyroscope. These data are quantified to obtain the accelerometer and gyroscope pulses. The satellite’s pseudo-range and pseudo-range rate as well as its position and velocity are obtained from the theoretical position, velocity, the attitude of the hypersonic vehicle’s 6DoF model output, and the global positioning system (GPS) satellite broadcast ephemeris. The simulation data can be used for the verification of the loose and tight coupling integrated navigation algorithms. The simulation test verifies the accuracy of the designed method.

## 1. Introduction

Near space is recognized as the altitude ranging 20–100 km, which is high for airplanes and too low for satellites. A hypersonic vehicle can travel Mach 5 (five times the speed of sound) or higher in near space [1,2]. Hypersonic vehicles employ strapdown inertial navigation systems (SINS) to obtain comprehensive navigation information, high autonomy, and a high update rate. The X-43A hypersonic vehicle employs an integrated navigation system with a SINS and a global positioning system (GPS) [3]. The X-51A navigation system is equipped with an inertial measurement unit (IMU) and a GPS receiver [4]. The Hypersonic Technology Vehicle 2 operates with a tightly coupled GPS/IMU system for guidance and approximately 3 m of error in the endo-atmospheric glide [5]. The navigation system of the German SHEFEX-2 hypersonic vehicle integrates an IMU, a GPS receiver, and a star tracker [6]. G. Hu et al present a new innovation orthogonality-based robust unscented Kalman filter (IO-RUKF) for INS/GNSS tightly coupled integrated navigation of hypersonic aircraft [7].The Hypersonic Conventional Strike Weapon utilizes a GPS/INS system for navigation and terminal guidance. Countries such as Russia, Japan, and India have also installed SINS/global navigation satellite system (GNSS) integrated navigation systems in their hypersonic vehicles [1]. 

A trajectory generator is a key component in the research and testing of the SINS/GNSS algorithm. Many approaches have been developed for the trajectory generator over the decades, which can be classified into three general categories [8]: (1) The first approach is based on pure mathematical models of SINS equations [9]. Profile generation (PROFGEN) [10] is the most traditional trajectory generator in this category, which can provide information on flight parameters such as position, velocity, attitude, angular velocity, and specific force. PROFGEN can support four flight maneuvers: vertical turns, horizontal turns, sinusoidal heading changes, and straight flight. (2) The second approach is based on actual flight data [8,11,12,13,14]. The simulated signals of gyroscopes and accelerometers are correlated not only with the vehicle’s kinematics and dynamic characteristics but also with the characteristics of post-processed GNSS’s pseudo-ranges and pseudo-range rate measurements. (3) The third approach is kinematic or dynamic models [15,16,17]; for example, the trajectory generator based on a six degree-of-freedom (6DoF) flight dynamics model and a flight control model. This is difficult to model because flight dynamics are highly correlated with the engine, aerodynamic data, mass, and actuator of the vehicles. 

The first and second approaches are limited in that the specific force and the angular velocity can only be derived from the equation of the inertial navigation system on the basis of a set flight profile in the first approach, while those in the second approach are post-processed. Although both the approaches are suitable for investigating the SINS/GNSS algorithm in digital simulation, they cannot be run in real time; therefore, they cannot be integrated with and adapted to the 6DoF flight dynamics model and flight control system in the real-time hardware-in-the-loop (HWIL) simulation. HWIL simulation for various hypersonic vehicles, including X-43A, has been complicated. For example, synchronization of the inertial navigation simulator with the HWIL simulation system took more than half a year for X-43A [15]. Traditional trajectory generators themselves cannot generate real trajectories. The first type of trajectory generator is similar to an open-loop system, and its trajectory data are generated by the mathematical model. The second type of trajectory generator is similar to a real-time closed-loop system, but its trajectory data are generated by a third party rather than its own system, for example, the simulation platform proposed in this article. In reference [15], the author proposed a magnetometer-augmented IMU simulator that takes the position and orientation of the carrier in the local frame as input and the sensor measurements as output, and considers error models including sensor dynamics, bias and white noise. In reference [17], the author introduced the development of a 6DoF simulation of a general hypersonic vehicle based on three different aerodynamic models, and completed digital simulation verification.

In this article, a trajectory generator of the SINS as a real-time closed-loop system is proposed that can be coordinated with the flight control system and the 6DoF model in the HWIL simulation. Moreover, it can generate the real aircraft trajectory based on real aerodynamic and engine data, and reproduce the real flying environment of the aircraft in the sky as realistically as possible. This article undertakes further research based on the reference, and the difference between this article and reference [16] is that on the basis of reference [16] the theoretical specific force and angular velocity will be integrated first, and then quantified into the number of pulses, which better simulates the real IMU. In addition, the GPS simulator is added to the simulation platform, and SINS/GPS integrated navigation system is formed with the SINS. Moreover, the position and velocity of a satellite as well as the pseudo-range and pseudo-range rate of a hypersonic vehicle are obtained from the 6DoF model and GPS satellite broadcast ephemeris. The simulation data can be used for the verification of the loose and tight coupling integrated SINS/GPS navigation algorithms, and it is applied in reference [18]. Compared with the two traditional trajectory generators, the trajectory generator based on flight mechanics introduced in this paper has been rarely studied in the existing literature, especially in combination with the semi-physical simulation of hypersonic aircraft. In addition, the trajectory generator has been actually applied in hypersonic vehicle experimental projects, and we have introduced the results of the semi-physical simulation of the hypersonic vehicle in reference [18].

## 2. Integrated Navigation Simulation Platform

Semi-physical simulation of aircraft is conducted using the HWIL simulation method, which is one of the important aspects in the development of aircraft. The flight control system of a hypersonic vehicle is connected with the HWIL simulation system to reproduce the flight environment of the hypersonic vehicle in air as realistically as possible in underground laboratory conditions and to verify and evaluate the performance indicators of the flight control system.

The overall architecture of HWIL simulation has been described in reference [16]. The 6DoF model [19] runs in the HiGale real-time simulator, which is a multi-model and multi-target real-time simulation platform that runs on National Instruments’ (NI) high-performance PXI (Peripheral Component Interconnection extensions for Instrumentation) controllers. HiGale is seamlessly connected with MATLAB, enabling Simulink to run in real time. The hardware interface is an NI multifunction reconfigurable I/O device, which is programmed for digital I/O, RS-422, and IMU pulses. The 6DoF model of a hypersonic vehicle includes 6DoF dynamics and kinematics models, aerodynamic models, mass/inertia models, Earth models, engine models, and guidance and control system models. The 6DoF model is the input source of the SINS in the HWIL simulation. The organic fusion of the 6DoF model and the SINS/GPS will enable the hypersonic vehicle guidance navigation and control system to conduct evaluation tests in the HWIL simulation system. On the basis of reference [16], a GPS simulator is added to the simulation platform to form a SINS/GPS integrated navigation system with the original inertial navigation system. Satellite-related data generated by GPS simulators can be used to verify the loose and tight coupling integrated SINS/GPS navigation simulation algorithms.

The HWIL simulation navigation simulator supports an IMU simulator and IMU model, a GPS simulator and GPS model, and a servo simulator (digital servo) and servo model, all in the loop mode. By using the IMU model and GPS model, system portability can be realized, which is highly beneficial for field test verification. These two models are described in detail below.

## 3. Inertial Measurement Unit (IMU) Model

The position, velocity, and attitude angle of the 6DoF model in the launch-centered Earth-fixed (LCEF) frame are the navigational theoretical parameters of a hypersonic vehicle. The LCEF frame is defined as: the coordinate origin is fixed to the launch point. The *x*-axis is in the horizontal plane of the launch point and points to the target direction; the *y*-axis is perpendicular to the horizontal plane of the launch point and points upward; and the *z*-axis and the x, *y*-axis form the right-handed coordinate system. The LCEF frame is usually represented by the *g* frame.

Details of the theoretical input of specific force and angular velocity, the IMU error model, and the velocity increment and angular increment of specific force and angular velocity in the 6DoF model are explained below.

**Figure 1:**
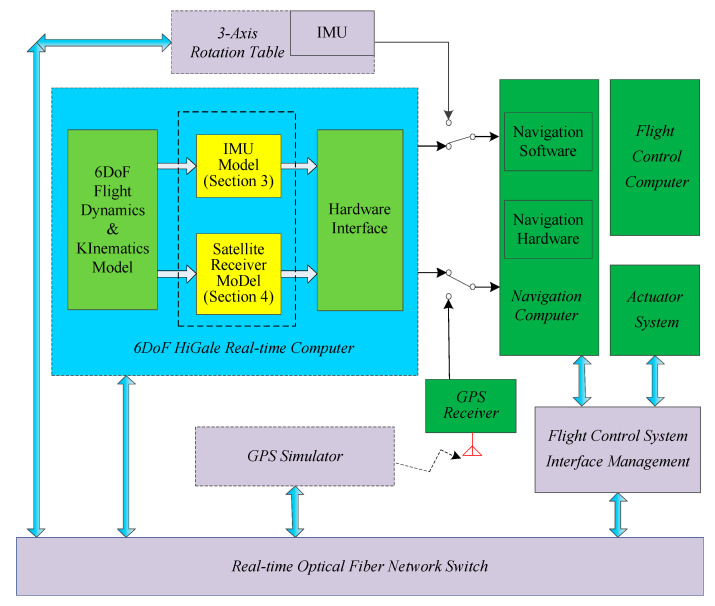
Illustration of the hardware-in-the-loop (HWIL) simulation system for hypersonic vehicle strapdown inertial navigation systems/global positioning system (SINS/GPS).

**Figure 2:**
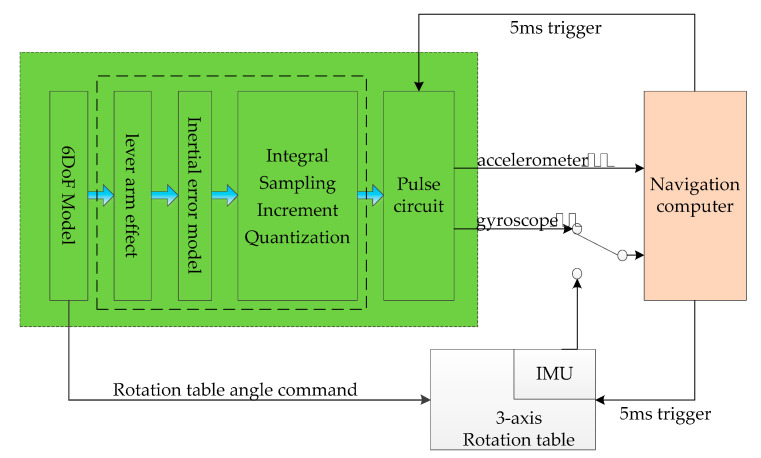
Strapdown inertial measurement unit (IMU) model.

Figure 1 indicates the location of the IMU model in the HWIL simulation system. Figure 2 presents the implementation of the IMU model. The specific force and angular velocity are obtained from a high-precision 6DoF model. Based on these input parameters, an IMU error model is included. Finally, the continuous IMU signal is subjected to integral sampling and quantization to obtain the IMU pulses.

### 3.1. Theoretical Input of Specific Force and Angular Velocity

The specific force $f^{b}$, which is the theoretical output of the accelerometer, is the sensitive force acting upon a unit mass other than gravity in the inertial frame; it is expressed as follows [16]:

(1) $$f^{b}=\left(P+R+F_{c}+F\prime_{k}\right)/m$$

Equation (1) indicates that the specific force obtained from the 6DoF model is a result of the combined action of the engine thrust P, aerodynamic force R, actuator control force $F_{c}$, and additional Coriolis force $F\prime_{k}$ of flight vehicles. The Coriolis force is a description of the offset of the linear motion of the mass point in the rotating system due to inertia relative to the linear motion of the rotating system, which comes from the inertia of the object motion [20]. The specific force reflects the actual motion state of a vehicle’s centroid, which is different from that in the traditional trajectory generator. 

In the 6DoF model, the angular velocity $\omega^{b}$ in the launch-centered inertial (LCI) frame is the theoretical output of the gyroscope. Equation (2) suggests that the angular velocity generated by the 6DoF model is a result of the combined action of all types of moment and it reflects the actual angular motion of the vehicle revolving about its centroid; this is different from the traditional trajectory generators. In the LCI frame, the dynamic equation of the centroid of the hypersonic vehicle is expressed as [20].

(2) $$I\cdot \frac{d\omega^{b}}{dt}+\omega^{b}\times (I\cdot \omega^{b})=M_{st}+M_{c}+M_{d}+M\prime_{rel}+M\prime_{k}$$

where I is the inertia tensor of the vehicle, $M_{st}$ is the aerodynamic stabilizing moment, $M_{c}$ is the control moment, $M_{d}$ is the damping moment, $M\prime_{rel}$ is the additional relative moment, and $M\prime_{k}$ is the additional Coriolis moment. The above moments is defined as follows [21]: during the flight of the aircraft, the point of action of the aerodynamic force does not coincide with the center of mass, so the aerodynamic force will form a rotational moment on the center of mass, and this moment is called the aerodynamic stability moment; by changing the thrust direction of the aerodynamic engine, the force and moment that control the flight of the aircraft are generated, and this moment is defined as the control moment; when the aircraft rotates relative to the atmosphere, the atmosphere will have a damping effect on it, and the acting moment is defined as the damping moment; the relative force and Coriolis inertial force generated by the relative flow of fuel in the aircraft create moments on the center of mass, which are defined as additional relative moment and the additional Coriolis moment. The detailed calculation formula can refer to reference [21]

### 3.2. Lever Arm Effects

The lever arm effect is a phenomenon, which means that when the rigid carrier has angular motion relative to the inertial space, the inertial sensors of the two inertial navigation systems at different positions will measure different specific forces, so that the calculated speed and position are also different [22]. In inertial navigation, the difference information of the main and sub inertial navigation system navigation parameters caused by the lever arm effect has nothing to do with the error propagation characteristics of the sub inertial navigation system. If it is not eliminated, it will affect the estimation accuracy of the error parameters of the sub-inertial navigation system and then affect the navigation performance [23].

**Figure 3:**
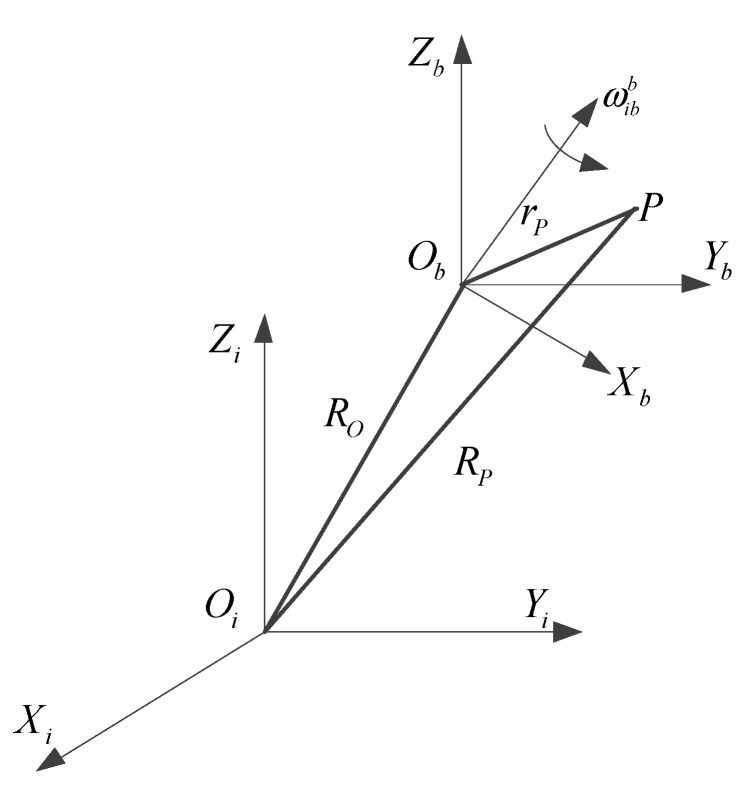
Schematic diagram of lever arm effect.

The schematic diagram of the lever arm effect is shown in Figure 3, *O_i_X_i_Y_i_Z_i_* is the inertial frame, *O_b_X_b_Y_b_Z_b_* is the carrier frame, *O_b_* is the swing center for carrier, $\omega_{ib}^{b}$ is the rotational angular velocity of the carrier relative to the inertial space. The accelerometer is installed at the fixed point *P* in the carrier frame; $R_{O}$ is the position vector of the origin of the carrier frame relative to the origin of the inertial frame; $R_{P}$ is the position vector of the point *P* relative to the origin of the inertial frame; $r_{P}$ is the position vector of the point *P* relative to the origin of the carrier frame.

Ideally, the installation point of the accelerometer should be located on the center of the carrier, that is the origin of the carrier frame *O_b_*, and when the mounting point *P* of the accelerometer deviates from the center of the carrier ($r_{P}$ is not zero). The lever arm effect error δf caused by $r_{P}$ can be expressed as:

$$\delta f={\left(\frac{d^{2}R_{P}}{dt^{2}}\right)}_{i}-{\left(\frac{d^{2}R_{O}}{dt^{2}}\right)}_{i}={\left(\frac{d^{2}r_{P}}{dt^{2}}\right)}_{b}+{\dot{\omega }}_{ib}^{b}\times r_{P}+\omega_{ib}^{b}\times {\left(\frac{dr_{P}}{dt}\right)}_{b}+2\omega_{ib}^{b}\times \left(\omega_{ib}^{b}\times r_{P}\right)$$

where the subscript *i* represents the differential between the relative and inertial frame; the subscript *b* represents the differential between the relative and the carrier frame. ${\left(\frac{dR_{O}}{dt}\right)}_{i}$ represents the linear velocity of the origin of the carrier frame relative to the inertial frame; ${\left(\frac{dR_{P}}{dt}\right)}_{i}$ represents the linear velocity of the point *P* relative to the inertial frame; ${\left(\frac{dr_{P}}{dt}\right)}_{b}$ represents the linear velocity of the point *P* relative to the carrier frame. ${\left(\frac{d^{2}R_{O}}{dt^{2}}\right)}_{i}$ is the specific force measured by the accelerometer at point *O_b_*, and ${\left(\frac{d^{2}R_{P}}{dt^{2}}\right)}_{i}$ is the specific force measured by the accelerometer at point *P*.

### 3.3. Gyroscope and Accelerometer Error Models

The gyroscope error model is given as follows:

(3) $$\delta \omega^{b}=B_{g}^{b}+M_{g}\omega^{b}+\varepsilon_{g}$$

(4) $$\left[\begin{array}{c} \delta \omega_{x}^{b}\\ \delta \omega_{y}^{b}\\ \delta \omega_{z}^{b} \end{array}\right]=\left[\begin{array}{c} B_{gx}\\ B_{gy}\\ B_{gz} \end{array}\right]+\left[\begin{array}{ccc} S_{gx} & M_{gxy} & M_{gxz}\\ M_{gyx} & S_{gy} & M_{gyz}\\ M_{gzx} & M_{gzy} & S_{gz} \end{array}\right]\left[\begin{array}{c} \omega_{x}^{b}\\ \omega_{y}^{b}\\ \omega_{z}^{b} \end{array}\right]+\left[\begin{array}{c} \varepsilon_{gx}\\ \varepsilon_{gy}\\ \varepsilon_{gz} \end{array}\right]$$

where $B_{g}^{b}$ is the gyroscope bias vector; $\omega^{b}$ is the angular velocity vector of the gyroscope; $M_{g}$ is the gyroscope misalignment error; $\varepsilon_{g}$ is the gyroscope random noise vector; and $S_{gi}$ is the gyroscope scale factor error, i=x,y,z.

The accelerometer error model is given as follows:

(5) $$\delta f^{b}=B_{a}^{b}+M_{a}f^{b}+D_{a}{\left(f^{b}\right)}^{2}+\varepsilon_{a}$$

(6) $$\left[\begin{array}{c} \delta f_{x}^{b}\\ \delta f_{y}^{b}\\ \delta f_{z}^{b} \end{array}\right]=\left[\begin{array}{c} B_{ax}\\ B_{ay}\\ B_{az} \end{array}\right]+\left[\begin{array}{ccc} S_{ax} & M_{axy} & M_{axz}\\ M_{ayx} & S_{ay} & M_{ayz}\\ M_{azx} & M_{azy} & S_{az} \end{array}\right]\left[\begin{array}{c} f_{x}^{b}\\ f_{y}^{b}\\ f_{z}^{b} \end{array}\right]+\left[\begin{array}{ccc} d_{ax} & 0 & 0\\ 0 & d_{ay} & 0\\ 0 & 0 & d_{az} \end{array}\right]\left[\begin{array}{c} {\left(f_{x}^{b}\right)}^{2}\\ {\left(f_{y}^{b}\right)}^{2}\\ {\left(f_{z}^{b}\right)}^{2} \end{array}\right]+\left[\begin{array}{c} \varepsilon_{ax}\\ \varepsilon_{ay}\\ \varepsilon_{az} \end{array}\right]$$

where $B_{a}^{b}$ is the accelerometer bias vector; $f^{b}$ is the accelerometer specific force; $M_{a}$ is the accelerometer misalignment error; $D_{a}$ is the error associated with the acceleration quadratic term; $\varepsilon_{a}$ is the accelerometer random noise vector; and $S_{ai}$ is the accelerometer scale factor error, i=x,y,z.

### 3.4. Implementation of Integral Quantification

**Figure 4:**
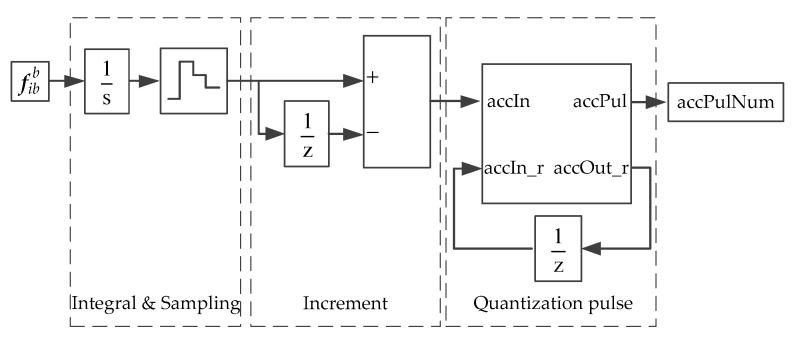
Implementation of velocity increment.

The specific force and angular velocity of the IMU are output in the form of velocity increment and angular increment, which are the integral results of the accelerometer theoretical value $f^{b}$ and the gyroscope theoretical value $\omega^{b}$ in unit time, respectively, as shown in Equations (8) and (9). The velocity increment and angular increment are further quantified and output as pulse numbers. Inside the IMU, the output quantification is generally performed after the high-frequency sampling process; that is, the IMU has the characteristics of internal high-frequency sampling and external low-frequency incremental output. In Figure 4, the acceleration output is taken as an example to illustrate the method of implementation of the velocity increment and the number of pulse.

(7) $$\Delta V=\int_{0}^{\tau }\left(f^{b}\left(\tau \right)+\delta f^{b}\right)d\tau$$

(8) $$\Delta \theta =\int_{0}^{\tau }\left(\omega^{b}\left(\tau \right)+\delta \omega^{b}\right)d\tau$$

As seen in Figure 4, through the sampling module, Simulink is divided into two parts, namely, 1 ms cycle period and 5 ms cycle period. For the 6DoF model, the 1 ms cycle is the simulation period (a smaller simulation period can be used according to the simulation accuracy need), and the 5 ms cycle is the running cycle of the simulation inertial device. Through the Simulink integral module, in the 1 ms cycle period, the acceleration/angular velocity information is accumulated every 1 ms within 5 ms, and the internal high-frequency sampling of the inertial device is simulated, so that there is no loss of the acceleration/angular velocity high-frequency information. In the 5 ms cycle period, the incremental output information of the inertial device is simulated by the Simulink delay block and the subtraction block. The incremental information of the IMU is quantified to obtain the quantified pulse number, and the implementation equations are obtained as follows:

(9) $$\left\{ \begin{array}{l} \Delta V_{Pul}=\left\lfloor \frac{\Delta V_{k}+\Delta V_{k-1}}{\mathrm{Pulse}\_\mathrm{Acc}}\right\rfloor \\ \Delta {V\prime }_{k}=\Delta V_{k}+\Delta V_{k-1}-\Delta V_{\mathrm{Pul}}\times \mathrm{Pulse}\_\mathrm{Acc} \end{array}\right.$$

where $\Delta V_{k}$ is the integral increment in the 5 ms cycle period, corresponding to accIn in Figure 4; $\Delta V_{k-1}$ is the margin after the quantification of the last period pulse, with the margin magnitude being less than 1 pulse equivalent, corresponding to accIn_r in Figure 4; $\Delta {V\prime }_{k}$ is the margin after the quantization of the current beat pulse, corresponding to accOut_r in Figure 4; $\Delta V_{\mathrm{Pul}}$ is the pulse number after current quantification, corresponding to accPul in Figure 4; PULSE_ACC is the pulse equivalent.

## 4. Satellite Receiver Simulation of Integrated Navigation

**Figure 5:**
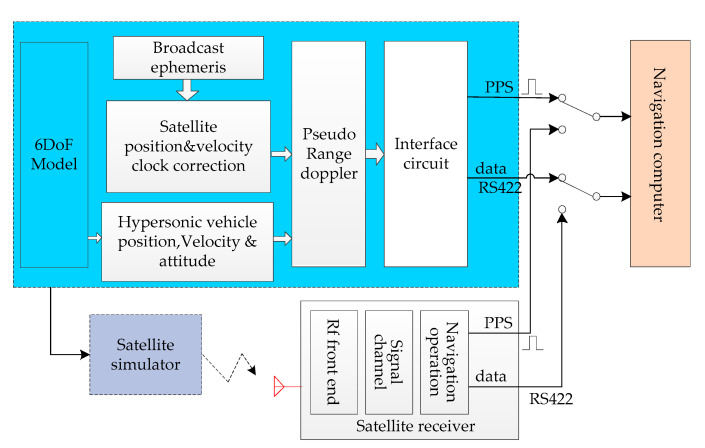
Satellite receiver model.

In the HWIL simulation diagram (Figure 1), the location of the GPS receiver model in the HWIL simulation is indicated. Figure 5 presents the implementation of the GPS receiver model. We specify the GPS satellite constellation through a GPS broadcast ephemeris file. The daily GPS broadcast ephemeris file (brdc) is obtained by merging individual site navigation files. The GPS broadcast ephemeris files are then used to generate the simulated pseudo-range and Doppler shift for the visible GPS satellites. Through the parameters provided by the ephemeris, the corresponding satellite parameters such as satellite position, speed, and clock correction can be calculated.

### 4.1. Satellite Receiver Simulation of Integrated Navigation

**Figure 6:**
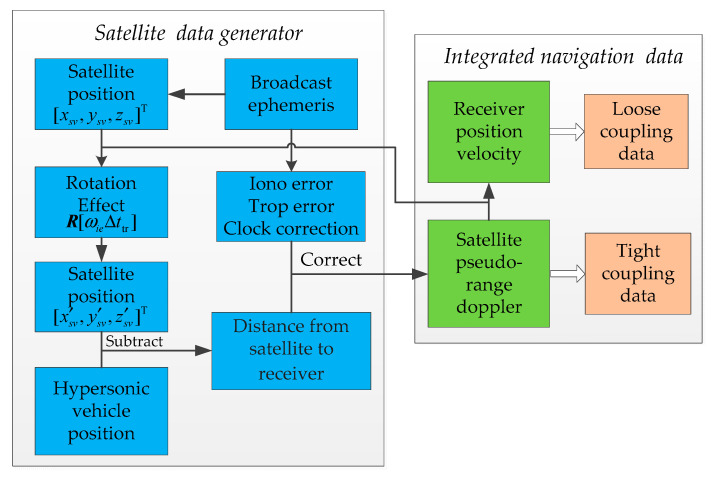
Satellite data simulation.

As shown in Figure 6, the steps of the simulation of satellite data in the loose and tight coupling integrated navigation are as follows. First, the GPS satellite ephemeris is used to calculate the position and velocity of the satellite; then, the theoretical position and velocity of the hypersonic vehicle 6DoF model and the position and velocity of the satellite are used to calculate the satellite’s pseudo-range and pseudo-range rate or Doppler shift, which are the simulation data required for tight coupling. Finally, using the combination of the position and velocity of the satellite, the position and velocity of the receiver are calculated by the least squares method, which are the simulation data required for loose coupling.

#### 4.1.1. Tight Coupling Data Simulation

Tight coupling SINS/GPS integrated navigation requires pseudo-range and pseudo-range rate measurements of the satellite. In the simulation platform, the pseudo-range and pseudo-range rate can be calculated from the position and velocity of the satellite and receiver. The calculation equation for the position and velocity of the satellite has been described in references [24,25].

The steps for the calculation of the pseudo-range and pseudo-range rate based on the position and velocity of the satellite and receiver are as follows:

(10) $$\left[\begin{array}{l} \Delta x\\ \Delta y\\ \Delta z \end{array}\right]=\left[\begin{array}{l} x_{sv}\\ y_{sv}\\ z_{sv} \end{array}\right]-\left[\begin{array}{l} x_{u}\\ y_{u}\\ z_{u} \end{array}\right]$$

Here, Δp=[Δx,Δy,Δz] is the position difference between the satellite and the receiver, ${\left[x_{u},y_{u},z_{u}\right]}^{T}$ is the position of the receiver in the Earth-centered Earth-fixed frame (ECEF, e frame), and ${\left[x_{sv},y_{sv},z_{sv}\right]}^{T}$ is the position of the satellite in the ECEF frame. The ECEF is defined as: the origin is the center of the Earth, and the x- and y-axes are in the equatorial plane of the Earth. The *x*-axis points to the prime meridian, while the *z*-axis is the Earth’s rotation axis [26].

(11) $$\Delta t_{tr}=\frac{\sqrt{\Delta x^{2}+\Delta y^{2}+\Delta z^{2}}}{c}$$

In Equation (12), $\Delta t_{tr}$ is the transmission time of the satellite signal from the satellite to the receiver, *c* is the speed of light.

(12) $$\left[\begin{array}{l} {x\prime }_{sv}\\ {y\prime }_{sv}\\ {z\prime }_{sv} \end{array}\right]=\left[\begin{array}{ccc} 1 & \;\omega_{ie}\Delta t_{tr} & 0\\ -\omega_{ie}\Delta t_{tr} & 1 & 0\\ 0 & 0 & 1 \end{array}\right]\left[\begin{array}{l} x_{sv}\\ y_{sv}\\ z_{sv} \end{array}\right]$$

${\left[{x\prime }_{sv},{y\prime }_{sv},{z\prime }_{sv}\right]}^{T}$ is the position coordinates of the satellite after considering the rotation effect of the earth, $\omega_{ie}$ is the earth’s rotation angular velocity.

(13) $$\left[\begin{array}{l} \Delta x\prime \\ \Delta y\prime \\ \Delta z\prime \end{array}\right]=\left[\begin{array}{l} {x\prime }_{sv}\\ {y\prime }_{sv}\\ {z\prime }_{sv} \end{array}\right]-\left[\begin{array}{l} x_{u}\\ y_{u}\\ z_{u} \end{array}\right]$$

$\Delta p\prime \;={\left[\Delta x\prime ,\Delta y\prime ,\Delta z\prime \right]}^{T}$ is the position difference between the satellite and the receiver after considering the rotation effect. Then, the approximate distance from the satellite to the receiver is:

(14) $$d=\sqrt{{\left(\Delta x\prime \right)}^{2}+{\left(\Delta y\prime \right)}^{2}+{\left(\Delta z\prime \right)}^{2}}$$

After considering the clock correction of the GPS, ionospheric error, and tropospheric error, the pseudo-range is obtained as:

(15) $$\rho =d+cT_{\mathrm{iono}}-c\delta t_{s}$$

Here, $T_{\mathrm{iono}}$ is the time delay caused by ionospheric errors, $\delta t_{s}$ is the satellite clock correction.

The pseudo-range rate is obtained as:

(16) $$\overset{\cdot }{\rho }\;=\frac{v_{x}\Delta x\prime \;+v_{y}\Delta y\prime \;+v_{z}\Delta z\prime }{\rho }$$

In Equation (17), $\left[v_{x},v_{y},v_{z}\right]$ is the velocity difference between the satellite and the receiver.

#### 4.1.2. Loose Coupling Data Simulation

Loose coupling requires the position and velocity of the satellite receiver, which are obtained from the position and velocity parameters of the satellite and the pseudo-range and pseudo-range rate (or Doppler shift) measurements.

The position and velocity of the receiver are calculated by the least squares method. The specific calculation steps are as follows.

The mathematical model of single-mode pseudo-range measurements is:

(17) $$\left\{\begin{array}{c} {\tilde{\rho }}_{1}\left(x_{u}\right)=\sqrt{{\left(x_{u}-x_{s_{1}}\right)}^{2}+{\left(y_{u}-y_{s_{1}}\right)}^{2}+{\left(z_{u}-z_{s_{1}}\right)}^{2}}+\mathrm{cb}+n_{\rho_{1}}\\ {\tilde{\rho }}_{2}\left(x_{u}\right)=\sqrt{{\left(x_{u}-x_{s_{2}}\right)}^{2}+{\left(y_{u}-y_{s_{2}}\right)}^{2}+{\left(z_{u}-z_{s_{2}}\right)}^{2}}+\mathrm{cb}+n_{\rho_{2}}\\ \vdots \\ {\tilde{\rho }}_{m}\left(x_{u}\right)=\sqrt{{\left(x_{u}-x_{s_{m}}\right)}^{2}+{\left(y_{u}-y_{s_{m}}\right)}^{2}+{\left(z_{u}-z_{s_{m}}\right)}^{2}}+\mathrm{cb}+n_{\rho_{m}} \end{array}\right\}$$

Equations (17) and (18) represents m pseudo-range measurements, $\left[x_{u},y_{u},z_{u}\right]$ is the position of the receiver, *b* is the deviation between the local clock of the receiver and GPST (global positioning system time); and $n_{\rho }$ is the error in the pseudo-range measurement. The known quantities ${\tilde{\rho }}_{i}$ are pseudo-range measurements and satellite coordinates $\left[x_{s_{i}},y_{s_{i}},z_{s_{i}}\right]$.

The pseudo-range measurement can be linearized and written in the form of a matrix as follows:

(18) $$\delta \rho_{m}=Hdx_{0}+n_{\rho_{m}}$$

In Equation (19), $\delta \rho ={\left[\delta \rho_{1},\delta \rho_{2},\cdots ,\delta \rho_{m}\right]}^{T}$, $H={\left[u_{1},u_{2},\cdots ,u_{m}\right]}^{T}$, $n_{\rho }={\left[n_{\rho_{1}},n_{\rho_{2}},\cdots ,n_{\rho_{m}}\right]}^{T}$, $u_{i}=\left[\frac{\delta \rho_{i}}{\delta x_{u}}{|}_{x_{0}},\frac{\delta \rho_{i}}{\delta y_{u}}{|}_{y_{0}},\frac{\delta \rho_{i}}{\delta z_{u}}{|}_{z_{0}},1\right]$, and $dx_{0}={\left[\left(x_{u}-x_{0}\right),\left(y_{u}-y_{0}\right),\left(z_{u}-z_{0}\right),\left(b-b_{0}\right)\right]}^{T}$.

The least squares estimation of Equation (18) is given by:

(19) $$dx_{0}={\left(H^{T}H\right)}^{-1}H^{T}\delta \rho$$

Equation (20) obtains the correction value between the initialization and the real point after the first power linearization; this correction value can be applied to update the initial point to obtain the corrected solution; that is:

(20) $$x_{1}=x_{0}+dx_{0}$$

Then, $x_{1}$ can be used as the starting point to repeat the process from linearization to Equation (21) in order to obtain a new correction value $dx_{1}$ and the previous solution is updated.

#### 4.1.3. Geometric Dilution of Precision

When performing satellite navigation solutions, the geometric dilution of precision can reflect the quality of satellite positioning to a certain extent. Therefore, the geometric dilution of precision is selected in the simulation platform as the index for evaluating the satellite signal generated by the satellite simulator [27,28].

According to the covariance matrix of the state error obtained by the least squares method in the previous section, the covariance matrix of the position error at this time can be obtained as follows:

(21) $$\mathrm{var}\left\{\delta x_{u}\right\}={\left(H^{T}H\right)}^{-1}H^{T}\mathrm{RH}{\left(HH^{T}\right)}^{-1}$$

Here, $\delta x_{u}$ is used to distinguish from the updated $dx_{i}$ of each iteration in the previous section; *R* is the covariance matrix of the noise vector in the pseudo-range measurement. It is generally assumed that the measurement noises of different satellites are independent of each other, so *R* is a diagonal matrix, expressed as $\mathrm{diag}\left\{\sigma_{1}^{2},\sigma_{2}^{2},\cdots ,\sigma_{m}^{2}\right\}$. Obviously, $\sigma_{i}^{2}$ is the noise power, which measures the quality of the *i*-th satellite pseudo-range measurement? $R=\sigma^{2}I$ is obtained and I can be a unit matrix m×m. Substituting $R=\sigma^{2}I$ in Equation (22), we obtain:

(22) $$\mathrm{var}\left\{\delta x_{u}\right\}={\left(H^{T}H\right)}^{-1}H^{T}\sigma^{2}\mathrm{IH}{\left(HH^{T}\right)}^{-1}=\sigma^{2}{\left(H^{T}H\right)}^{-1}$$

Let ${\left(H^{T}H\right)}^{-1}$ be written with $\left[h_{i.j}\right]$, where $h_{i.j}$ represents the *i*-th row and *j*-column elements of the matrix. Hence,

(23) $$\left\{\begin{array}{c} \mathrm{var}\left\{\delta x_{u}\right\}=\sigma^{2}h_{1,1}\\ \mathrm{var}\left\{\delta y_{u}\right\}=\sigma^{2}h_{2,2}\\ \mathrm{var}\left\{\delta z_{u}\right\}=\sigma^{2}h_{3,3}\\ \mathrm{var}\left\{\delta b\right\}=\sigma^{2}h_{4,4} \end{array}\right\}$$

It can be seen from Equation (24) that the elements on the diagonal of the ${\left(H^{T}H\right)}^{-1}$ matrix reflect the accuracy of the positioning results. Therefore, the dilution of precision of the satellite receiver is given as follows:

Position dilution of precision $\left(\mathrm{PDOP}\right)=\sqrt{h_{1,1}+h_{2,2}+h_{3,3}}$

Time dilution of precision $\left(\mathrm{TDOP}\right)=\sqrt{h_{4,4}}$

Geometric dilution of precision $\left(\mathrm{GDOP}\right)=\sqrt{h_{1,1}+h_{2,2}+h_{3,3}+h_{4,4}}$

Horizontal positional dilution of precision $\left(\mathrm{HDOP}\right)=\sqrt{h_{1,1}+h_{2,2}}$

Vertical positional dilution of precision $\left(\mathrm{VDOP}\right)=\sqrt{h_{3,3}}$

The dilution of precision can be seen as a linear mapping from the measurement error to the state estimation error. In the case where the measurement errors are the same, a larger dilution of precision may result in a larger state estimation error, and a smaller dilution of precision results in a considerably smaller state estimation error. It can be seen from the definition of the dilution of precision that it is independent of the actual measurement noise, while it can only be related to ${\left(H^{T}H\right)}^{-1}$, which is directly calculated from the ***H*** matrix.

### 4.2. Global Positioning System (GPS) Error

The flying height of hypersonic vehicles is more than 20 km. The GPS errors to be considered mainly include ionospheric errors and satellite clock corrections.

#### 4.2.1. Ionospheric Error

The ionosphere is a layer of the Earth’s atmosphere that is ionized by the solar ray; it is 50–1000 km from the surface of the Earth and the inner boundary of the Earth’s magnetosphere [27,28,29]. Under the intense illumination of sunlight, the neutral gas molecules in the ionosphere are ionized, resulting in the generation of a large number of positive ions and free electrons. These positive ions and free electrons affect the propagation of navigational waves.

When the GPS satellite signal passes through the ionosphere, a certain delay exists, which is proportional to the number of free electrons encountered [30,31]. The electron density is affected by factors such as local time, geomagnetic latitude, and sunspot cycle.

The ionospheric correction model used is the Klobuchar ionospheric delay model [32]. The Klobuchar model abstracts ionospheric delay as a cosine function. *A* is the amplitude of the cosine curve; *PER* is the period of the cosine curve. The composition of the ionospheric correction algorithm for the GPS receiver requires the receiver’s approximate latitude and longitude positions $\left(\phi_{U},\lambda_{U}\right)$ as well as the azimuth and elevation angles (Az,El) relative to each satellite.

**Figure 7:**
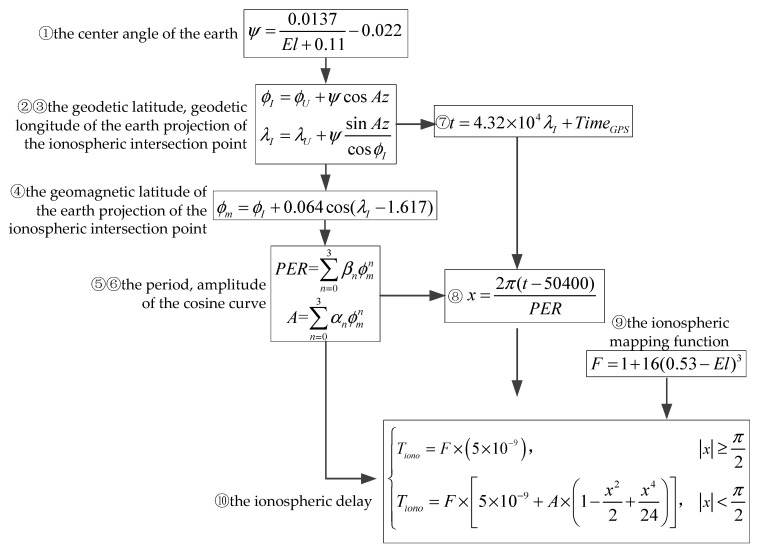
The calculation steps of the ionospheric delay.

The calculation steps are shown in Figure 7.

In Figure 7, ψ is the center angle of the Earth, $\phi_{I}$, $\lambda_{I}$, and $\phi_{m}$ are the geodetic latitude, geodetic longitude, and geomagnetic latitude of the earth projection of the ionospheric intersection point, respectively, *t* is the local time of the ionospheric intersection point, *F* is the ionospheric mapping function, $T_{\mathrm{iono}}$ is the ionospheric delay. When *A* is smaller than 0, *A* equal to 0 is taken, when *PER* is smaller than 72,000, *PER* equal to 72,000 is taken, $\alpha_{i}$, $\beta_{i}$ is the ionospheric parameters of the *i*-th satellite in the satellite navigation message.

#### 4.2.2. Satellite Clock Correction

A precise clock is critical in GPS. The GPS receiver receives signals from satellites whose transmission time is proportional to the pseudo-range measurement. Then, the relationship between the transmission time and pseudo-range measurement is:

(24) $$t_{r}=\frac{\rho }{c}$$

Here, $t_{r}$ is the propagation time of the signal from the satellite to the receiver, ρ is the pseudo-range measurement, and *c* is the speed of light.

The satellite emission signal time can be expressed as:

(25) $$t_{\mathrm{sv}}=t_{m}-t_{r}$$

Here, $t_{\mathrm{sv}}$ is the satellite signal transmission time and $t_{m}$ is the signal reception time.

The satellite clock error can be calculated from the polynomial factors obtained from the GPS navigation message.

(26) $$\Delta t_{\mathrm{sv}}=a_{f_{0}}+a_{f_{1}}\left(t-t_{\mathrm{oc}}\right)+a_{f_{2}}{\left(t-t_{\mathrm{oc}}\right)}^{2}$$

Here, $\Delta t_{\mathrm{sv}}$ is the satellite clock correction value, $a_{f_{0}}$ is the satellite clock error, $a_{f_{1}}$ is the small error, $a_{f_{2}}$ is the small frequency drift, $t_{\mathrm{oc}}$ is the clock reference point, and the value of $\left(t-t_{\mathrm{oc}}\right)$ should be corrected within seconds of a week.

Then, the correction value of the signal propagation time is:

(27) $$t=t_{\mathrm{sv}}-\Delta t_{\mathrm{sv}}-\Delta t_{r}$$

Here, $\Delta t_{r}$ is a relativistic correction value, which can be expressed as:

(28) $$\Delta t_{r}=-4.442807633\times 10^{-10}e\sqrt{a}\sin E_{k}(s)$$

Here, *e* is the eccentricity of the satellite orbit, *a* is the semi-major axis length, and $E_{k}$ is the eccentric anomaly.

The satellite clock difference can be defined as:

(29) $$\delta t_{s}=\Delta t_{\mathrm{sv}}+\Delta t_{r}$$

The correction equation for the pseudo-range measurement is:

(30) $$\rho_{\mathrm{corrected}}=\rho_{\mathrm{measured}}+c\delta t_{s}$$

In the above parameters,$a_{f_{0}}$,$a_{f_{1}}$,$a_{f_{2}}$, *e*, *a*, and $E_{k}$ are obtained from the broadcast ephemeris.

## 5. Simulation Verification

The simulation verification used the 1100 s trajectory data of the hypersonic vehicle to verify the simulation. The initial state of the trajectory is shown in Table 1.

**Figure 8:**
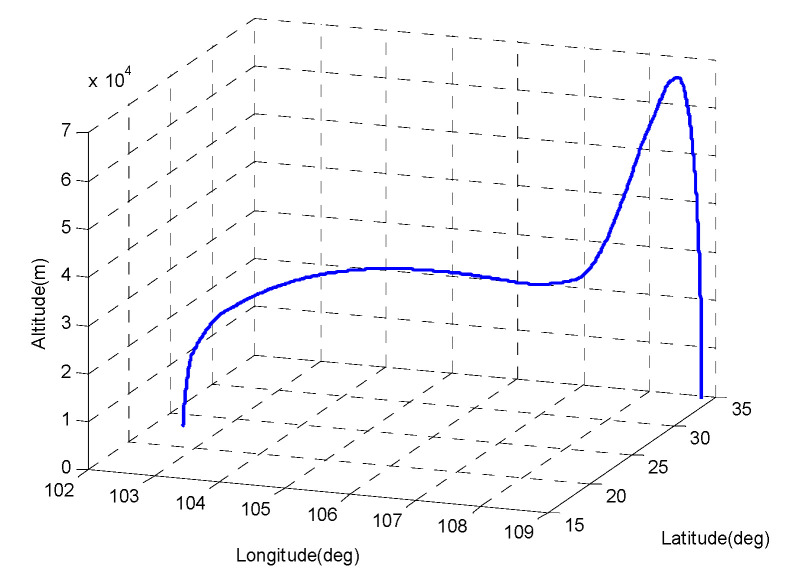
Simulated flight profile of a hypersonic vehicle.

The typical trajectory of a hypersonic boost-glider is presented in Figure 8 [18,33].

### 5.1. IMU Data Simulation

**Figure 9:**
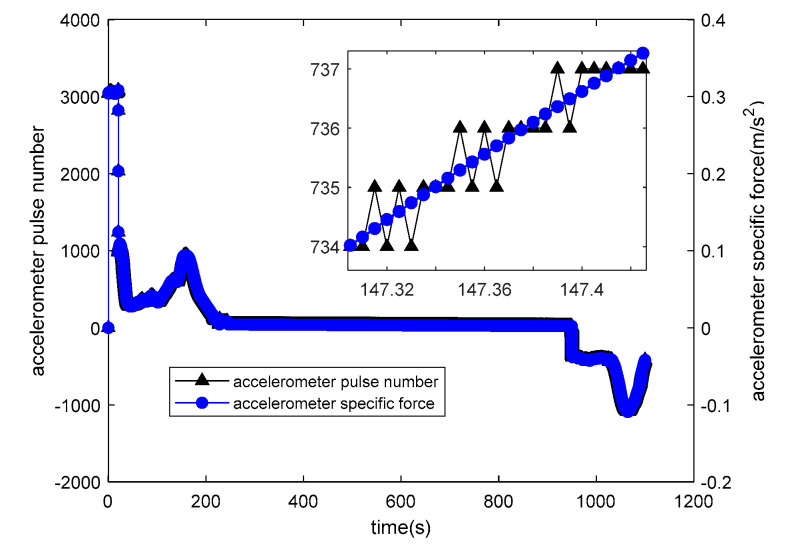
Output of accelerometer pulse number and accelerometer specific force.

**Figure 10:**
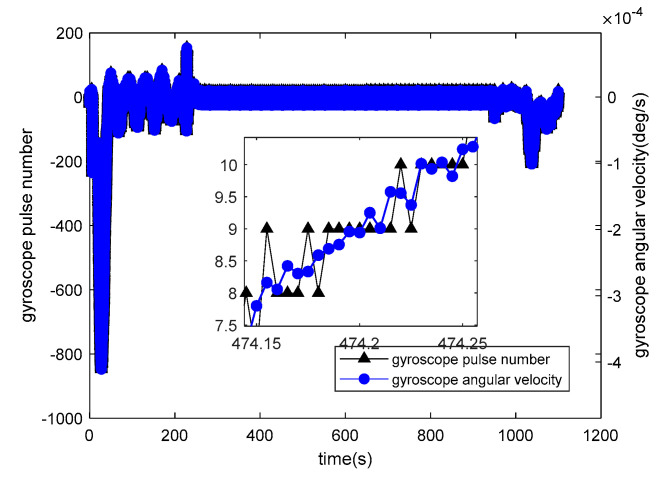
Output of gyroscope pulse output and gyroscope angular velocity.

The relationship between the number of accelerometer pulses and the specific force is depicted in Figure 9. 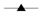 is the output of the accelerometer pulse number. Due to the quantification process, the number of pulses is an integer; 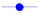 is the accelerometer specific force. Accelerometer pulse equivalent is 0.0001 $m/s^{2}$. The relationship between the number of gyroscope pulses and the angular velocity is depicted in Figure 10. 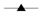 is the output of the gyroscope pulse number and 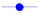 is the gyroscope angular velocity. Gyroscope pulse equivalent 0.1×(π/180)/3600rad/s.

### 5.2. GPS Receiver Data Simulation

**Figure 11:**
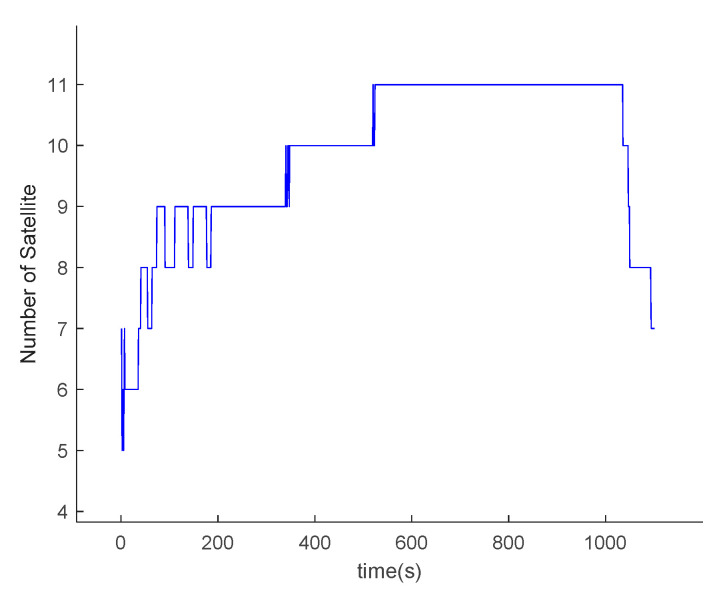
Number of visible satellites.

The data of the trajectory generator are imported into the GPS simulator to obtain corresponding GPS satellite data. Since the attitude of the hypersonic vehicle changes constantly during the actual flight, it may affect the satellite receiver’s ability to determine the visible satellites. As shown in Figure 11, the number of visible satellites varies.

**Figure 12:**
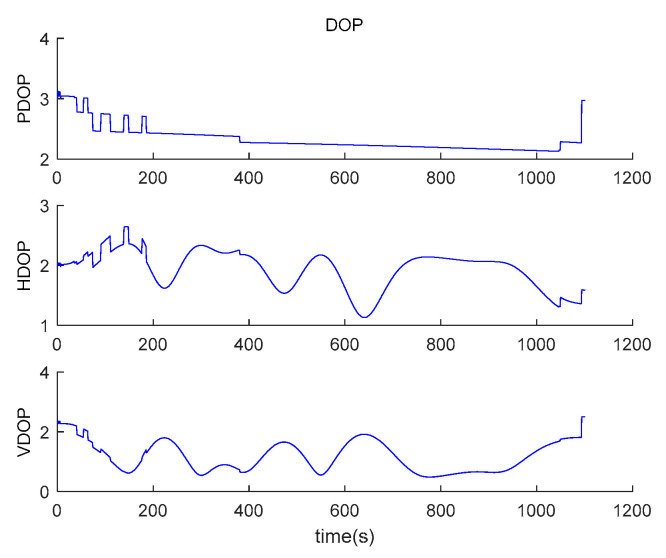
Positional dilution of precision of satellites.

The positional dilution of precision of satellites is shown in Figure 12.

**Figure 13:**
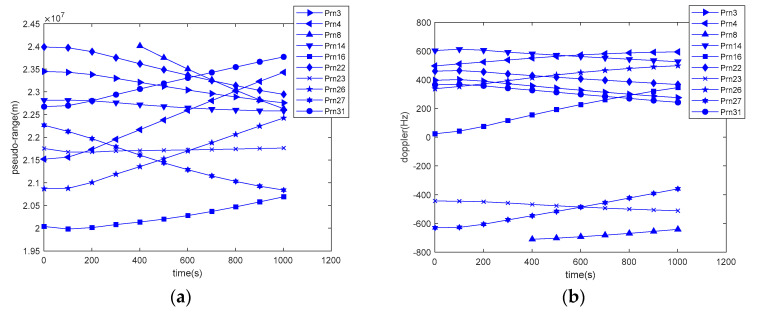
The variation of tight coupling data: (**a**) satellite pseudo-range variation; (**b**) satellite Doppler shift.

Satellite data can be divided into tight and loose coupling data. Tight coupling data include satellite pseudo-range and pseudo-range rate (Doppler shift); Figure 13 shows the variation of these parameters.

**Figure 14:**
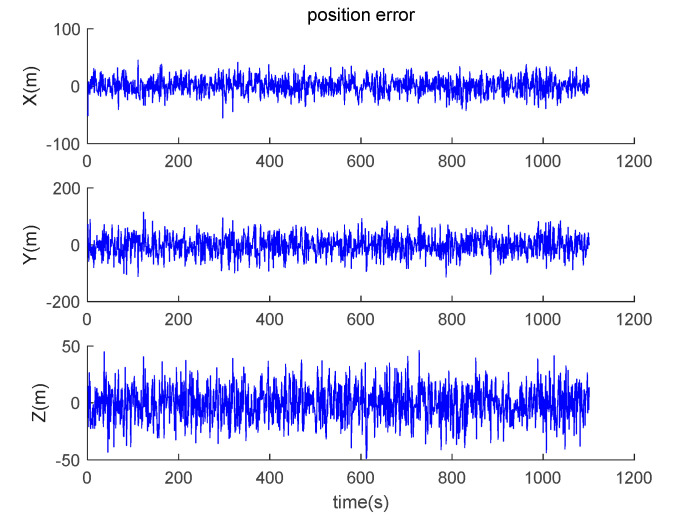
Position errors between theoretical trajectory and calculated trajectory.

**Figure 15:**
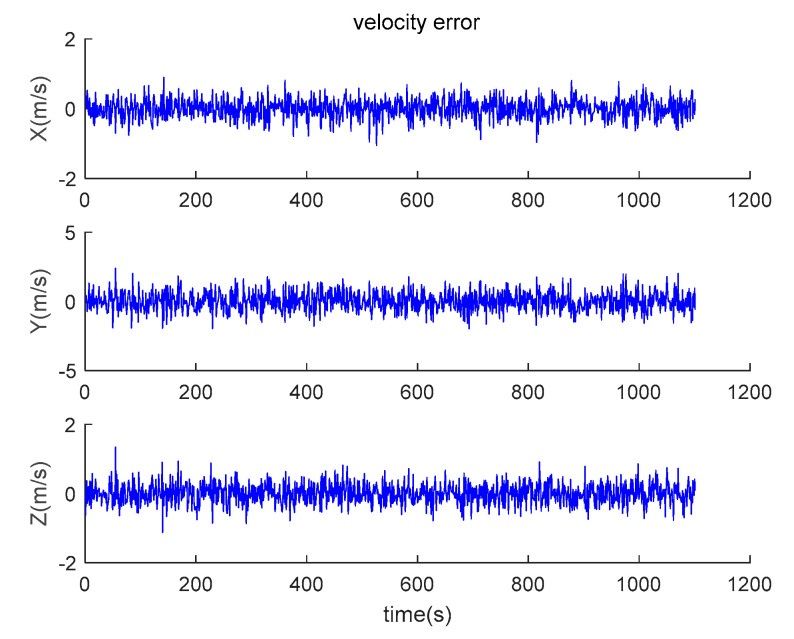
Velocity errors between theoretical trajectory and calculated trajectory.

The data output from the satellite simulator is input to the satellite receiver for verification, and the position and velocity errors between the original trajectory and the calculated trajectory are obtained as shown in Figure 14 and Figure 15.

It can be seen from the simulation results that the position errors are within 20 m (horizontal direction) and velocity errors are within 0.5 m/s (vertical direction). According to the calculation, the root mean square errors of the position are 14.9438, 36.6367 and 15.9965, respectively; the root mean square errors of the velocity are 0.2821, 0.6998 and 0.3042, respectively.

### 5.3. Strapdown Inertial Navigation Systems (SINS)/GPS Receiver Data Simulation

In the simulation verification of the integrated navigation algorithm, the gyroscope constant drift [34] is 3°/h(1σ), the gyroscope random error is $0.3{}^{\circ}/\sqrt{h}(1\sigma )$, the gyroscope scale factor error is $100\times 10^{-6}$, the accelerometer constant bias is $1\times 10^{-3}g(1\sigma )$; the accelerometer random error is $1\times 10^{-4}g/\sqrt{Hz}(1\sigma )$, the accelerometer scale factor error is $100\times 10^{-6}$; the initial roll angle, yaw, pitch errors are 60″, 20″, 20″, the initial velocity error is 0.05 m/s, the initial position is 5 m [18,33].

**Figure 16:**
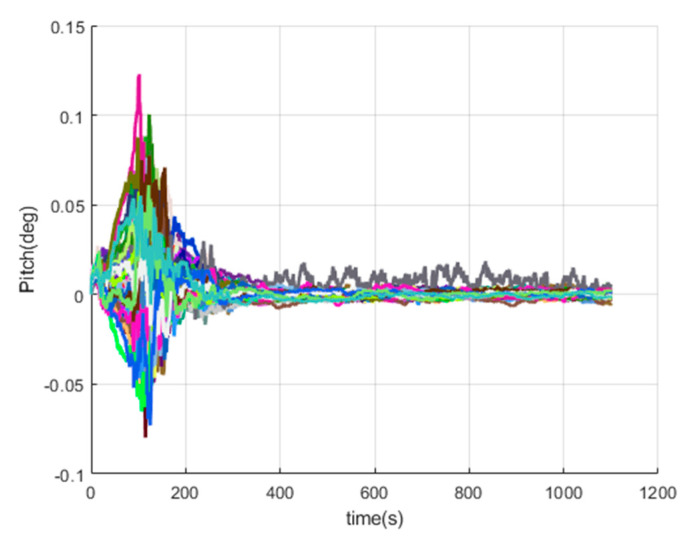
The pitch angle errors of SINS/GPS in the launch-centered Earth-fixed (LCEF) frame.

**Figure 17:**
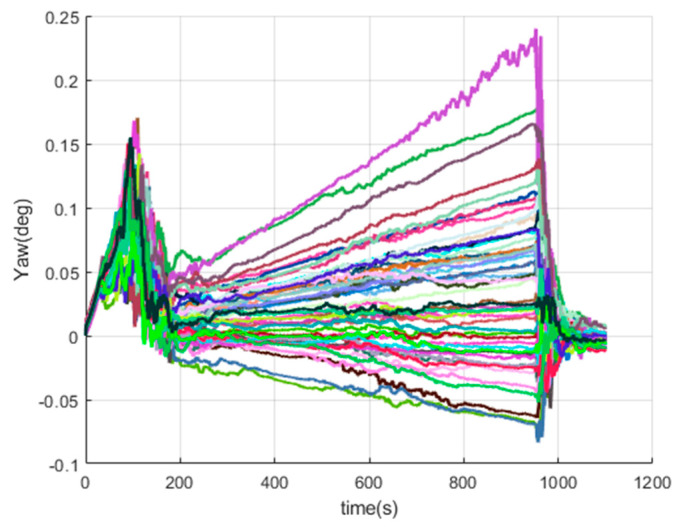
The yaw errors of SINS/GPS in the LCEF frame.

**Figure 18:**
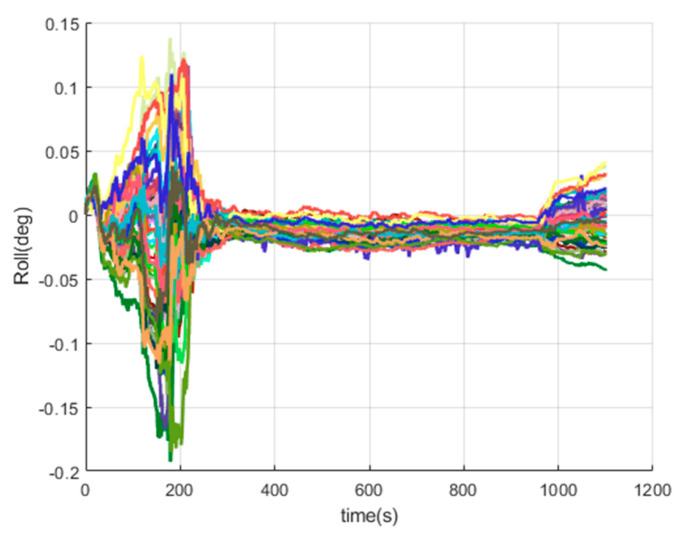
The roll errors of SINS/GPS in the LCEF frame.

**Figure 19:**
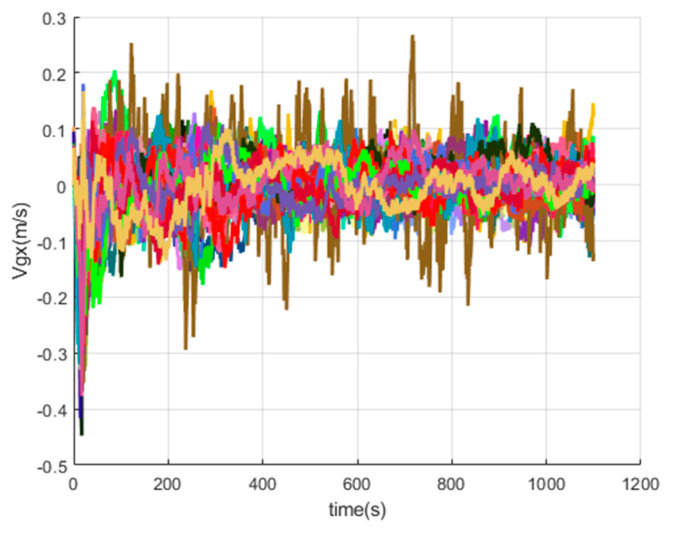
The *x*-axis velocity errors of SINS/GPS in the LCEF frame.

**Figure 20:**
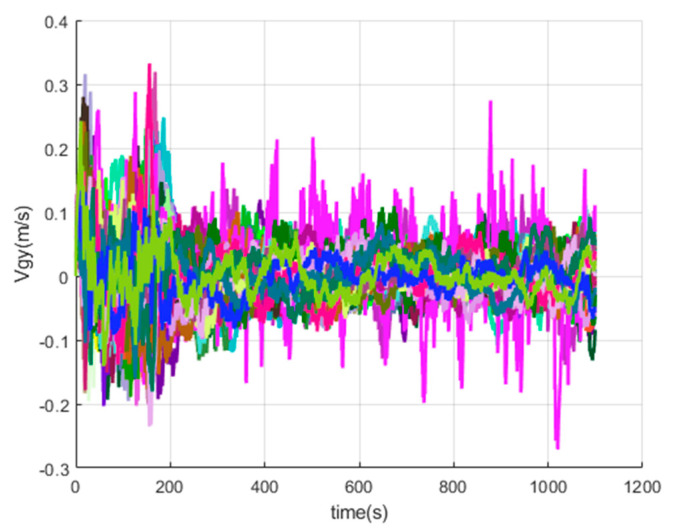
The *y*-axis velocity errors of SINS/GPS in the LCEF frame.

**Figure 21:**
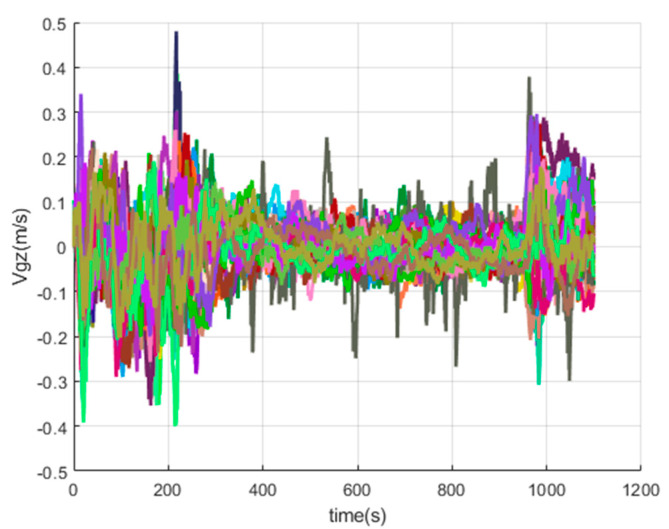
The *z*-axis velocity errors of SINS/GPS in the LCEF frame.

**Figure 22:**
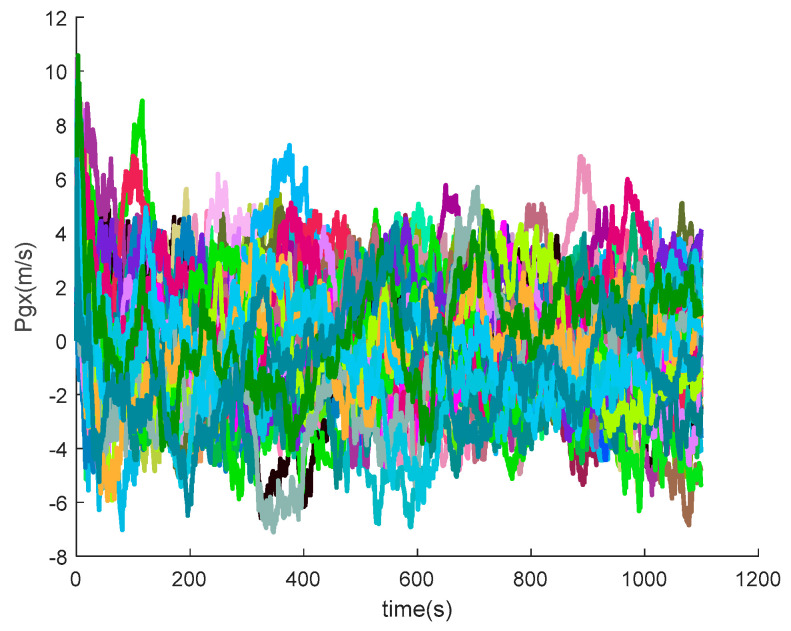
The *x*-axis position errors of SINS/GPS in the LCEF frame.

**Figure 23:**
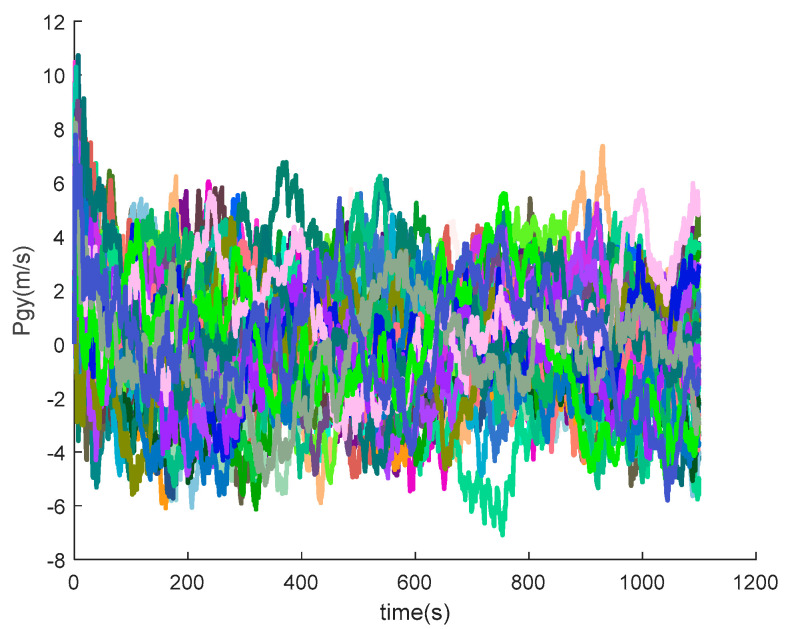
The *y*-axis position errors of SINS/GPS in the LCEF frame.

**Figure 24:**
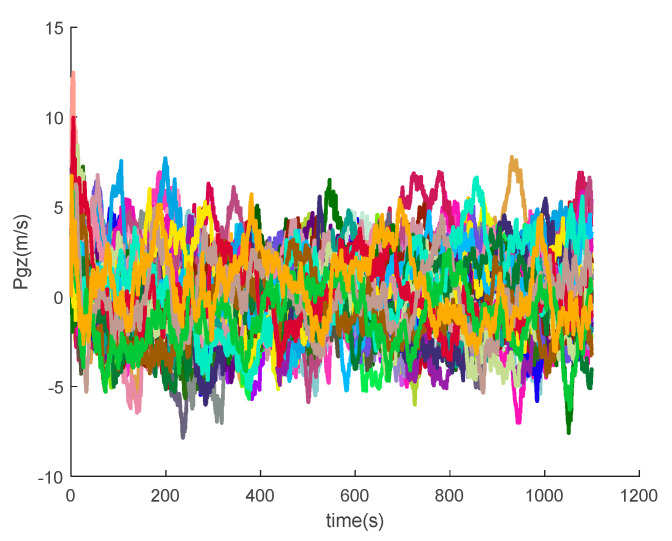
The *z*-axis position errors of SINS/GPS in the LCEF frame.

**Figure 25:**
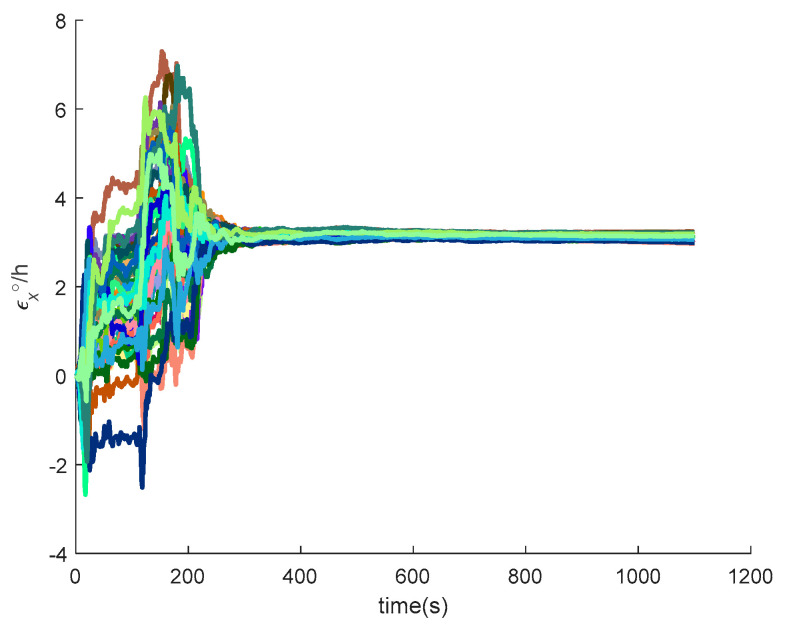
The *x*-axis gyroscope constant drift of SINS/GPS in the LCEF frame.

**Figure 26:**
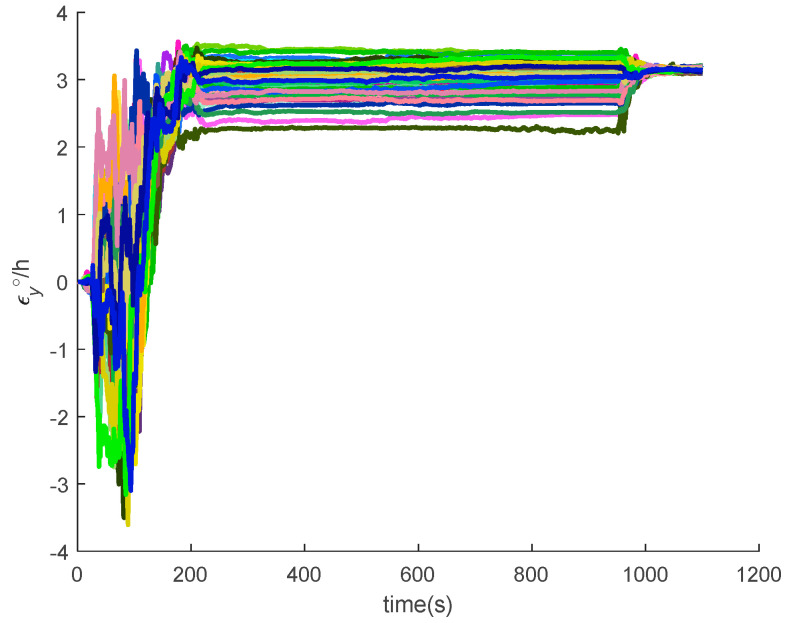
The *y*-axis gyroscope constant drift of SINS/GPS in the LCEF frame.

**Figure 27:**
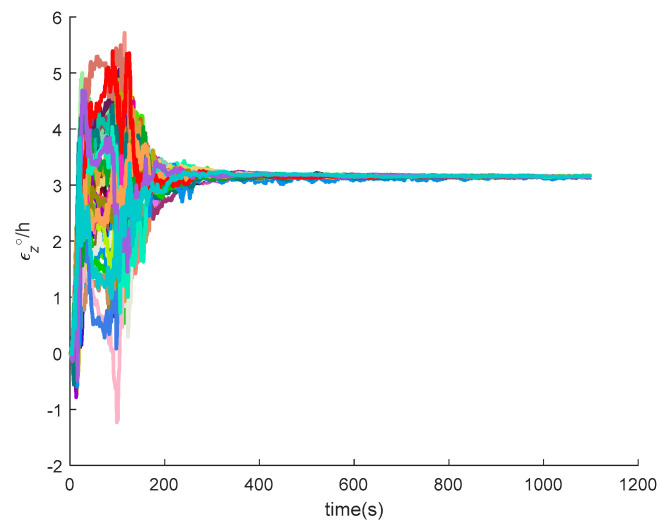
The *z*-axis gyroscope constant drift of SINS/GPS in the LCEF frame.

**Figure 28:**
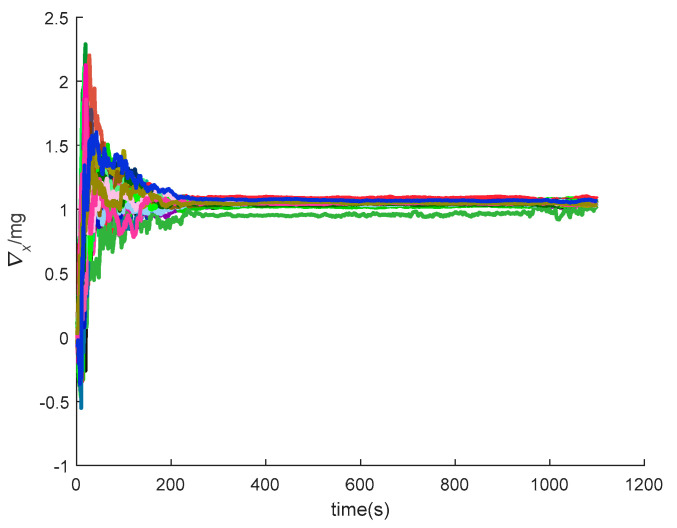
The *x*-axis accelerometer constant bias of SINS/GPS in the LCEF frame.

**Figure 29:**
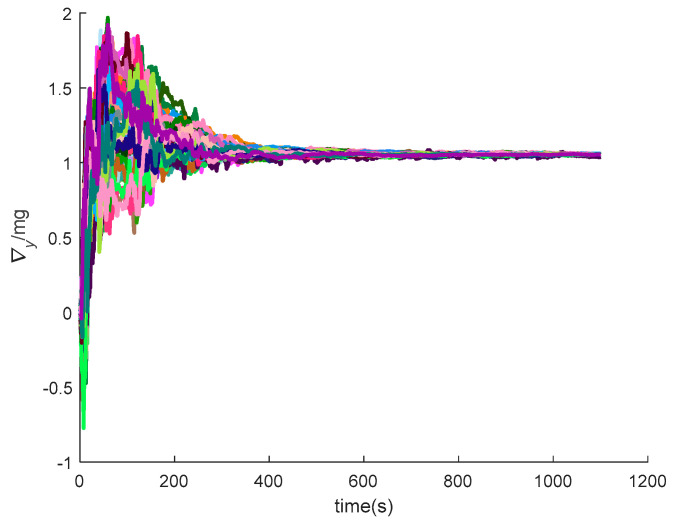
The *y*-axis accelerometer constant bias of SINS/GPS in the LCEF frame.

**Figure 30:**
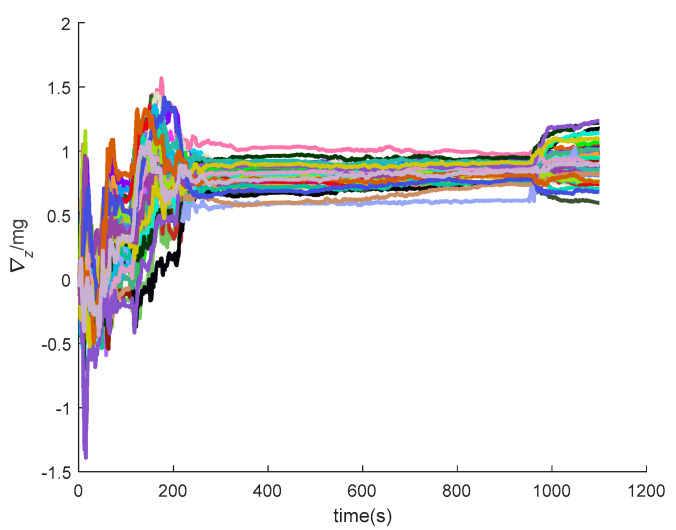
The *z*-axis accelerometer constant bias of SINS/GPS in the LCEF frame.

Figure 16, Figure 17, Figure 18, Figure 19, Figure 20, Figure 21, Figure 22, Figure 23, Figure 24, Figure 25, Figure 26, Figure 27, Figure 28, Figure 29 and Figure 30 present the simulation results of the SINS/GPS integrated navigation system conducted 50 times. The difference between them is that random errors are added to the gyroscope constant drift and accelerometer constant bias to better simulate the real inertial navigation system. Note that the three attitude angle errors of the integrated navigation system can basically converge to within 0.05°; the speed error in three directions can reach 0.2 m/s; and the position error of the direction is within 10 m; the gyroscope constant drift can converge to 3°/h; the accelerometer constant bias can converge to $1\times 10^{-3}g$. This result meets the navigation requirements of hypersonic vehicles.

### 5.4. Time Analysis

**Figure 31:**
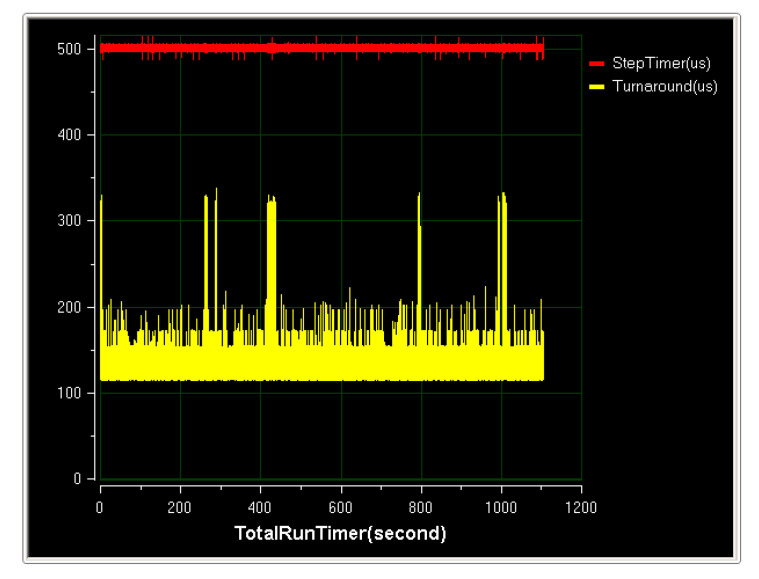
HiGale real-time simulator real-time simulation.

The results of the HiGale real-time simulator real-time simulation are shown in the Figure 31. In the figure, TotalRunTimer is the system running time, the unit is second, StepTimer is the HiGale simulation step size, the unit is microsecond, and Turnaround is the simulation software time consumption, the unit being the microsecond. This article uses a 1 ms simulation cycle, but HiGale internally uses the fourth Runge–Kutta method, that is, using a 500 μs cycle, Turnaround is the simulation software running time, both are less than 500 μs.

## 6. Conclusions

The simulation platform for the inertial navigation algorithm of hypersonic vehicles based on flight mechanics proposed in this paper is different from the traditional trajectory generator. It can generate a real aircraft trajectory, reproduce the real flying environment of the aircraft in the sky as realistically as possible, and can be coordinated with the flight control system in the HWIL simulation to verify and evaluate the performance index of the flight control system. The simulation platform can also generate simulation data to verify the accuracy of the integrated navigation algorithm. The simulation test in this paper verifies the correctness of the design method.

## Figures and Tables

**Table 1:** The initial state of the trajectory.

Parameters	Value
latitude	34.2°
longitude	108.9°
height	400 m
velocity	0 m/s
launch angle	200°
pitch angle	90°
roll angle	0°
yaw angle	0°
